# Correspondence between Aortic and Arterial Stiffness, and Diastolic Dysfunction in Apparently Healthy Female Patients with Post-Acute COVID-19 Syndrome

**DOI:** 10.3390/biomedicines11020492

**Published:** 2023-02-08

**Authors:** Cristina Tudoran, Felix Bende, Renata Bende, Catalina Giurgi-Oncu, Raluca Dumache, Mariana Tudoran

**Affiliations:** 1 Department VII, Internal Medicine II, Discipline of Cardiology, University of Medicine and Pharmacy “Victor Babes” Timisoara, E. Murgu Square, Nr. 2, 300041 Timisoara, Romania; 2 Center of Molecular Research in Nephrology and Vascular Disease, Faculty of Medicine, University of Medicine and Pharmacy “Victor Babes” Timisoara, E. Murgu Square, Nr. 2, 300041 Timisoara, Romania; 3 County Emergency Hospital “Pius Brinzeu”, L. Rebreanu, Nr. 156, 300723 Timisoara, Romania; 4 Academy of Romanian Scientists, Ilfov Str. Nr. 3, 50085 Bucuresti, Romania; 5 Department VII, Internal Medicine II, Discipline of Gastroenterology, University of Medicine and Pharmacy “Victor Babes” Timisoara, E. Murgu Square, Nr. 2, 300041 Timisoara, Romania; 6 Center of Advanced Research in Gastroenterology and Hepatology, Faculty of Medicine, University of Medicine and Pharmacy “Victor Babes” Timisoara, 300041 Timisoara, Romania; 7 Department VIII, Neuroscience, Discipline of Psychiatry, University of Medicine and Pharmacy “Victor Babes” Timisoara, E. Murgu Square, Nr. 2, 300041 Timisoara, Romania; 8 Department VIII, Discipline of Forensic Medicine, University of Medicine and Pharmacy “Victor Babes” Timisoara, E. Murgu Square, Nr. 2, 300041 Timisoara, Romania; 9 Center for Ethics in Human Genetic Identification, University of Medicine and Pharmacy “Victor Babes” Timisoara, E. Murgu Square, Nr. 2, 300041 Timisoara, Romania

**Keywords:** COVID-19, endothelial dysfunction, arterial stiffness, aortic elasticity, diastolic dysfunction

## Abstract

(1) Background: Abnormally increased arterial and aortic stiffness (AS and AoS), which are often associated with diastolic dysfunction (DD), represent common alterations in COVID-19. In this study, we aimed to assess, by transthoracic echocardiography (TTE) and pulse-wave velocity (PWV), the frequency of these dysfunctions in patients with post-acute COVID-19 syndrome and to highlight potential correlations between their severity and multiple clinical and laboratory parameters. (2) Methods: In total, 121 women were included in our study, all of whom were younger than 55 and had been diagnosed with post-COVID-19 syndrome. Of those women, 67 also had metabolic syndrome (MS) (group A), whereas the other 54 did not (group B); 40 age-matched healthy subjects were used as controls (group C). (3) Results: Patients in group A had worse values of indexes characterizing AS and AoS and had more frequent DD compared to those from group B and group C (*p* < 0.0001). The statistical analysis evidenced significant associations between these indexes and the time that had elapsed since COVID-19 diagnosis, the factors that characterize the severity of the acute disease and those that specify MS. Multivariate regression analysis identified the following as the main independent predictors for DD: values of the AoS index, the C-reactive protein, and the triglyceride–glucose index. (4) Conclusions: Altered AS, AoS, and DD are common in patients with post-COVID-19 syndrome, especially with concurrent MS, and these parameters are apparently associated not only with the severity and time elapsed since COVID-19 diagnosis but also with MS.

## 1. Introduction

**Figure 1:**
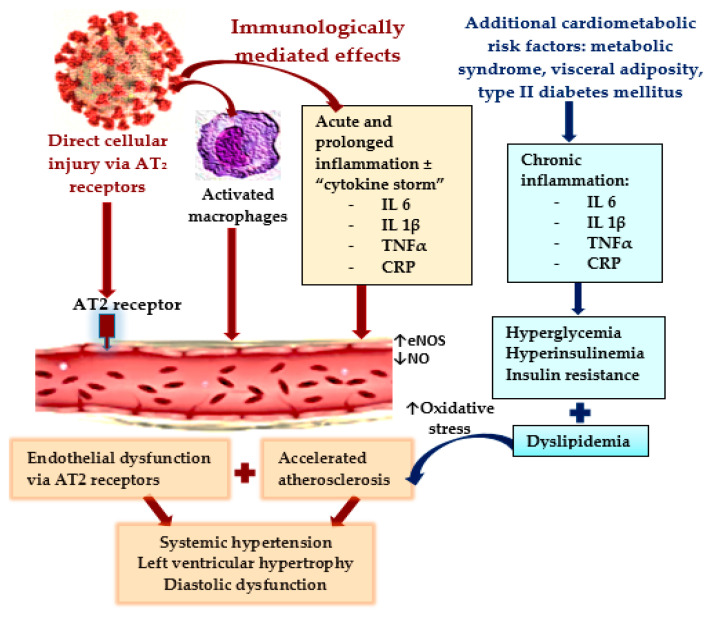
Pathophysiological mechanisms responsible for endothelial dysfunctions and their consequences in COVID-19.

As infections with the severe acute respiratory syndrome coronavirus 2 (SARS-CoV-2) increased at the beginning of the year 2020 into an alarming pandemic, with increased morbidity and mortality, it became obvious that the coronavirus disease (COVID-19) not only affects the lungs but is actually also a multisystem disease, potentially even with long-term consequences, because approximately one quarter of infected people go on to develop post-COVID-19 syndrome during recovery [1,2,3,4]. As this disease has continued to evolve for nearly 3 years, the pathophysiological pathways through which the SARS-CoV-2 virus affects the human organism have become progressively apparent [5,6]. Its direct action, by binding to the angiotensin-converting enzyme 2 (ACE2) receptors from the surface of the cellular wall, resulting in their downregulation, has been described since the early stages [7,8]. Nevertheless, the immunologically mediated effects from an over-activation of innate and adaptive immunity—which result in an increased release of acute phase reactants, such as pro-inflammatory cytokines, especially interleukine-6 (IL-6), interleukine-1β (IL-1β), ferritin, and C-reactive protein (CRP), together with the activation of macrophages—play a crucial role in the development of multiple systemic injuries. It is worth mentioning that COVID-19 also precipitates the development of β-cell injury and insulin resistance (IR) through the release of IL-1β and tumor necrosis factor α (TNF-α) [7,9], as shown in Figure 1. Therefore, in individuals already suffering from metabolic dysfunctions (MD), which are known to be associated also with an increased pro-inflammatory state and multiple metabolic alterations, among which IR plays a crucial role, the supplementary inflammatory burden triggered by SARS-CoV-2 infection frequently predisposes patients to the development of multisystem complications [9,10,11,12]. Other than the deleterious effects on the respiratory system, COVID-19 is often associated with a large spectrum of cardiovascular complications, which may persist during the recovery from this illness, causing multiple cardiac dysfunctions [13,14,15,16]. Most of these cardiovascular complications are consequences of the direct effects of the virus on the myocytes and endothelial cells (mediated via the ACE2 receptors), but especially, of the inflammation-modulated injuries (cytokine-release syndrome) [7]. Endothelial dysfunction has been identified as one of the most precocious and frequent alterations during COVID-19, which result from multiple and interconnected pathophysiological pathways, as shown in Figure 1. 

Several studies have discussed that the SARS-CoV-2 virus has an increased inotropism for capillaries and small vessels, resulting in the elevation of arterial stiffness (AS) [7,17,18]. MD, which is characterized by IR and/or even metabolic syndrome (MS), both of which are frequently encountered in patients recovering from COVID-19, can precipitate AS [8]. The downregulation of ACE2 receptors causes a reduction in arterial vasodilation, followed by increased systemic vascular resistance, AS, and higher blood pressure (BP) values, resulting in an elevation of the afterload. This elevation, in turn, causes the development of left ventricular hypertrophy (LVH) and the occurrence of diastolic dysfunction (DD), as well as elevated pressure in the left atria (LAP) and pulmonary veins, leading to heart failure with preserved ejection fraction (HFpEF) [4,19,20].

Because AS, which is measured either in the aorta or the peripheral arteries, has emerged as a surrogate marker for vascular aging and atherosclerosis, independently predicting an increased risk for adverse cardiovascular events, one may be tempted to also estimate this risk in individuals suffering from post-acute COVID-19 syndrome. Moreover, AS can be assessed non-invasively by pulse wave velocity (PWV), which is currently the best-validated diagnostic method, with a high accuracy and reproducibility. It is inversely correlated with vascular compliance and predicts cardiovascular outcomes. On the other hand, several indexes reflecting aortic elasticity, the left ventricular LV mass index (LVMI) defining LVH, and the parameters required to characterize DD and appreciate cardiac performance can be easily ascertained and monitored by transthoracic echocardiography (TTE) [21,22,23,24].

The aim of this study was to ascertain, by non-invasive methods, the frequency and severity of AS, impaired aortic elasticity, and DD in female patients with and without MS who are currently suffering from post-acute COVID-19 syndrome, in comparison with age-matched healthy controls. Another aim was to research whether, in these patients, there is an association between an elevated AS, impaired aortic elasticity and DD, and several other factors related to an antecedent SARS-CoV-2 infection, and/or elements characterizing MS. 

## 2. Materials and Methods

### 2.1. Study Population

Our study was carried out on a group of 121 female patients suffering from post-acute COVID-19 syndrome, who were examined in the internal medicine, cardiology, and psychiatry ambulatory services of the County Clinical Emergency Hospital of Timisoara, between September 2021 and June 2022, for persisting non-characteristic complaints, the most frequent being dyspnea, persistent cough, unexplained and long-lasting fatigue, reduced effort capacity, tachycardia, chest pain, increased BP values, insomnia, vertigo, concentration difficulties, and memory impairments. On the premises that gender differences concerning the normal values and prevalence of LVMI, AS, aortic elasticity factors, and DD elements would influence our results, only female patients were included in our study. On the other hand, as it is a well-known fact that AS is strongly affected by numerous hormonal factors, we decided to select only premenopausal women without a personal history of chronic diseases and with, therefore, an adequate pre-existing health status, (specifically, without an established diagnosis of cardiovascular or chronic renal diseases or type 2 diabetes mellitus (T2DM)). All patients were selected with the requirement that they had been non-smokers for at least one year before becoming infected with the SARS-CoV-2 virus. 

From an initial population of 235 women who were younger than 55 years old and who had been diagnosed with post-acute COVID-19 syndrome, based on a thorough physical examination, a further 53 were diagnosed with significant previously unknown pathologies, some of which were deemed to be complications of the SARS-CoV-2 infection, and were, thus, considered unsuitable for our project. The remaining 182 female patients were offered a more detailed medical examination, including TTE, electrocardiogram (ECG), and laboratory tests, if they were willing to participate in our project and if they met the required inclusion/exclusion criteria. This was confirmed by a signed informed consent. 

In order to prove previous SARS-CoV-2 virus infection, all patients were requested to provide a positive result of real-time reverse transcriptase–polymerase chain reaction (RT-PCR) assay of nasal and pharyngeal swabs, alongside either a discharge summary or an ambulatory assessment, lab tests, pulmonary radiography, or chest computed tomography scan (CCT) and an ECG. Additionally, the patients were also required to provide any recent medical documents containing physical exam, ECG, and TTE results (even abbreviated) or laboratory data (a minimum requirement was lipid panel, basal (fasting) blood glucose (BBG), and uric acid level) attesting their health condition. These results allowed us to confirm the existence of any metabolic dysfunctions at baseline. A total of 138 subjects agreed to take part in our study and could provide the necessary documents. They also fulfilled the following inclusion and exclusion criteria, which are presented in Table 1.

A control group of 40 healthy, premenopausal women of similar age was collected, which comprised health care workers and individuals who attended the ambulatory services of our hospital for regular check-ups required for driver’s license medical evaluations and occupational health and who were willing to sign the informed consent and to follow procedures of our study.

### 2.2. Procedures of the Study and Clinical and Laboratory Examinations

After receiving the informed consent, we gathered the available medical information regarding the course of the acute SARS-CoV-2 infection and the most recent assessments of their health status from all 138 participants. Firstly, we evaluated the severity and consequences of COVID-19 based on individual medical records that contained the results of the chest radiography or CCT scans, along with a description of the extent of their lung injury, an ECG, and blood tests. Subsequently, we focused on the analysis of their pre-COVID-19 health status, and several clinical and laboratory parameters of interest for this study, such as the presence of chronic diseases (those with current therapies for systemic hypertension or T2DM were excluded from our study, but occasionally elevated or borderline values of blood pressure or BBG were accepted), and possible references to risk factors, body weight and height, BP values, and ECG and TTE results (even if these were only mentioned as normal). Afterwards, an ECG, PWV assessment, and a detailed TTE were performed in all participants to identify significant cardiovascular alterations that could have been missed at the previous evaluations. In all patient groups, these assessments were repeated after 6 months.

#### 2.2.1. Assessment of AS

After the measurement of systolic (SBP) and diastolic BP (DBP), the PWV was assessed in patients and controls with a SphygmoCor device (AtCor Medical, Sydney, NSW, Australia) at the level of the right carotid and of the right femoral artery. The PWV was calculated by determining the time interval necessary for the pulse wave to progress along the arterial segment between the two sites [25,26,27]. The measurements were performed in the morning, in basal conditions, after 10 min of rest in a quiet room.

#### 2.2.2. Echocardiographic Examination 

Afterwards, a comprehensive TTE was performed in both patients and controls in accordance with guideline recommendations [23,24]. From the view of the parasternal long and short axes, we performed the conventional measurements of heart structures with considerations for the parietal contractility and continued to assess the LV mass index (LVMI) to establish the presence of an LVH, which is defined by an LVMI of over 95 g/m^2^ in women. From the apical 4-chamber view, we assessed the LAVI, considering volumes superior to 34 mL/m^2^ to be indicative of left atrial hypertrophy. To appreciate the aortic elasticity, we determined by TTE in M-mode from a transthoracic parasternal long-axis view, the diastolic and systolic diameters of the ascendant aorta, at 3–4 cm above the insertion of the aortic valve, at the moment of the maximum aortic anterior expansion, and at the peak of the QRS complex, respectively. Subsequently, we calculated the following parameters:

Aortic distensibility (AoD) = (2 × strain)/(SBP − DBP);

Aortic strain (AoS) = (systolic diameter − diastolic diameter)/diastolic diameter;

and

Aortic stiffness index β (AoSI) = ln(SBP/DBP)/strain (where ln = natural logarithm).

We continued with the study of the LV and right ventricle (RV) functions and the assessment of DD, also recording the existence of other cardiac abnormalities.

To estimate the existence of DD, we used the criteria stated by Nagues [24] and, by using pulsed Doppler, recorded from an apical 4-chamber view the diastolic mitral inflow at the level of the mitral valve annulus, measured the peak velocity of the early diastolic wave (E) and the peak velocity of the late diastolic wave (A), and calculated the E/A ratio. From the same view, at the level of the septal and lateral mitral annulus, tissue Doppler imaging (TDI) was employed to register the early diastolic velocity (e’) and late diastolic velocity (a’), and, afterwards, average values of these velocities and the E/e’ ratio were calculated. Again from an apical window, at the level of tricuspid valve, we employed continuous wave Doppler to register the maximal velocity of the tricuspid regurgitation (TRV). The last criterion for framing DD was LAVI, which was assessed as explained above. A type I DD was defined by an E/A ratio of over 0.8, with an E velocity of under 50 cm/s, whereas a type III DD was confirmed by an E/A ratio of over 2. In case of an E/A ratio under ≤0.8 but with an E of over 50 cm/s, or if the E/A was between 0.8 and 2, a type II DD was considered if two of the following criteria were present: an average E/e’ ratio of over 14, LAVI greater than 34 mL/m^2^, and/or a TRV higher than 2.8 m/s. If only one criterion was present, we diagnosed a type I DD [24].

#### 2.2.3. Laboratory Assessments 

As a consequence of these assessments, a further 17 patients were excluded from our study due to previously unidentified significant medical conditions. The remaining 121 subjects and controls underwent an assessment of their BMI and waist circumference (WC), followed by blood sample collection to determine BBG, serum creatinine with the calculation of eGFR, uric acid, total cholesterol (Tchol), low-density lipoprotein (LDLchol) cholesterol, high-density lipoprotein (HDLchol) cholesterol, triglyceride (TG), and C-reactive protein (CRP) levels. Several indexes that are considered to be significant for the evaluation of MS were calculated, as follows:

The triglyceride–glucose (TyG) index represents the logarithm of the product of BBG and fasting TG, and the formula is ln[BBG (mg/dL) × TG (mg/dL)/2]. The TyG index has been recommended as an alternative indicator for the IR because of its correlation to lipotoxicity and glucotoxicity [28,29]. A close relationship has been previously demonstrated between the TyG and cardiometabolic outcomes, T2DM, endothelial dysfunction, systemic hypertension, cardiovascular diseases, stroke and, more recently, with the health outcomes from COVID-19 [28,30,31]. In the medical literature, the normal cut-off values reported for TyG vary widely, between 4 and 8 (due to the position of 2 in the TyG index formula [28,32]. 

The lipid accumulation product (LAP), which is accepted as an indicator for visceral adiposity, was calculated based on the WC and fasting TG. The formula for women is LAP = (WC (cm) − 58) × TG (mmol/L). The reference cut-off values for LAP range from 25.16 to 31.59 cm × mmol/L for women. LAP is largely employed as an indicator for MS and abdominal obesity and is considered to be a risk factor for cardiovascular diseases, and it predicts adverse cardiovascular events [31,33]. 

The visceral adiposity index (VAI) was calculated based on the following formulas: VAI = (WC (cm)/(36.58 + (BMI × 1.89) × (TG/0.81) × (1.52/ HDL) for women [32,34].

To quantify the physical consequences of an infection with the SARS-CoV-2 virus based on the number of persisting symptoms and to evaluate the rehabilitation process, we employed the post-COVID-19 functional status (PCFS) scale. This tool was created to quantify the amplitude of functional limitations. Based on this scale, the absence of symptoms/limitations is quoted as 0; discreet limitations of quotidian activities associated with few symptoms are quantified as 1; slight limitations, but with more significant symptoms, are represented by a 2; moderate limitations, which are associated with the inability to perform prior current activities due to persistent symptoms, but retaining the ability to take care of themselves without assistance are quantified as a 3; severe physical limitations due to severe symptoms, requiring care, are represented by a 4 [35]. 

The Local Scientific Research Ethics Committee of the County Emergency Hospital from Timisoara, Romania, approved our study (Nr. 206/07.2020 and Nr. 297/11.04.2022).

### 2.3. Statistical Analysis

Data analysis was performed by employing the MedCalc Version 19.4 (MedCalc Software Corp., Brunswick, ME, USA) and Microsoft Office Excel 2019 (Microsoft for Windows). Descriptive statistical methods were used for the patients’ clinical data. We utilized the Kolmogorov–Smirnov test to classify the distribution of numerical variables and, as a result, continuous variables with normal distribution were presented as mean and standard deviations (SD). Those with non-normal distribution were presented as median and associated interquartile range (IQRs), whereas categorical variables were expressed as counts (percentages). To compare the three groups (A, B, C), we used the Kruskal–Wallis H test, followed by a post-hoc analysis with the Mann–Whitney U test with a Bonferroni correction applied. To compare continuous variables with normal distribution, we utilized the Student’s *t*-test, and for variables with non-normal distribution, the Mann–Whitney U-test was applied. To highlight the potential relationship between parameters characterizing AS, AoS, AoSI, AoD, and DD and multiple other demographic, anthropometric, echocardiographic, and laboratory findings, we employed the Spearman’s rank–order correlation and the Pearson’s χ^2^-test for categorical variables group analysis. Several uni- and multivariate regression analyses were applied to evaluate independent predicting factors associated with the risk of developing AS, impaired aortic elasticity, and DD. The Akaike information criteria were employed to obtain the best regression model. We considered a *p*-value of less than 0.05 to be indicative of statistical significance.

## 3. Results

The objective of this study was to analyze the results of clinical and laboratory assessments performed in a population of 121 women aged between 34 and 55 years, with a mean age of 47.72 ± 7.15 years, suffering with post-acute COVID-19 syndrome in comparison to a control sample of 40 healthy women who are of similar ages (between 38 and 55 years, mean age of 49.47 years) and who reported not to have ever suffered from a SARS-CoV-2 infection. Their clinical and biological parameters are depicted in Table 2. 

Both patients and controls were premenopausal, had no history of cardiovascular diseases, and were non-smokers or reported that they had given up smoking for over a year. Although none of the patients was diagnosed as suffering from metabolic dysfunctions, at a thorough baseline evaluation, we noticed that 67 of them met the criteria for MS. Thus, we subdivided them into group A, which consisted of 67 patients with MS and post-acute COVID-19 syndrome, and group B, which consisted of 54 subjects with complaints typical of post-COVID-19 syndrome. Although some of the patients had one or even two defining elements, they failed to meet at least three MS diagnostic criteria. We researched for significant differences between groups A and B, and surprisingly, the statistical analysis revealed significant disparities between the samples with regard to all clinical and laboratory-studied parameters, in most cases with *p* < 0.0001. More specifically, the patients with MS had more severe cases of COVID-19; presented with more extended lung injury (*p* = 0.0002); had more complaints and worse PCFS scores (*p* < 0.0001), having been diagnosed sooner with post-acute COVID-19 syndrome; and had worse laboratory results. We proceeded to analyze the differences between groups B and C, and, although the controls were somewhat older than the women in group B (*p* = 0.0126), there was no difference concerning their BMI, WC, BBG, and LDL chol. However, we noted statistically significant differences regarding the remaining analyzed parameters (Table 2), namely, the BP values, heart rate (HR), HDLchol. (*p* < 0.0001), triglycerides, and uric acid levels, as well as the TyG index (*p* < 0.0001), LAP, and VAI (*p* < 0.0001), although the laboratory results were within normal limits in both groups (Table 2). 

**Figure 2:**
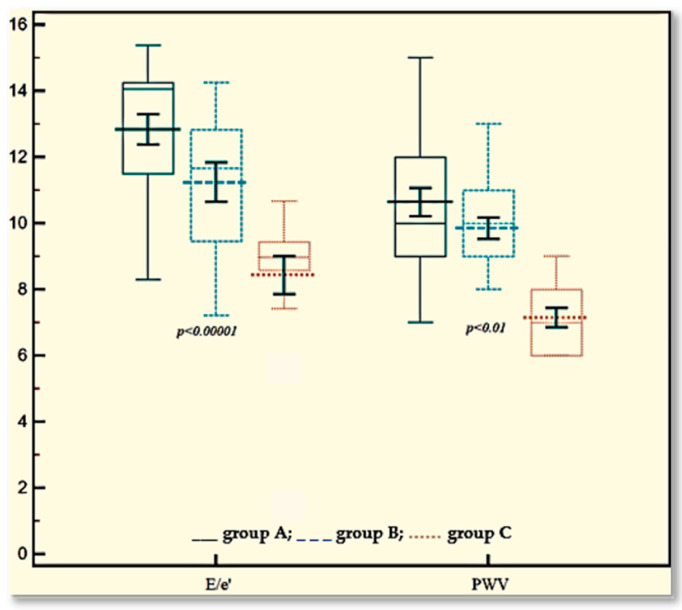

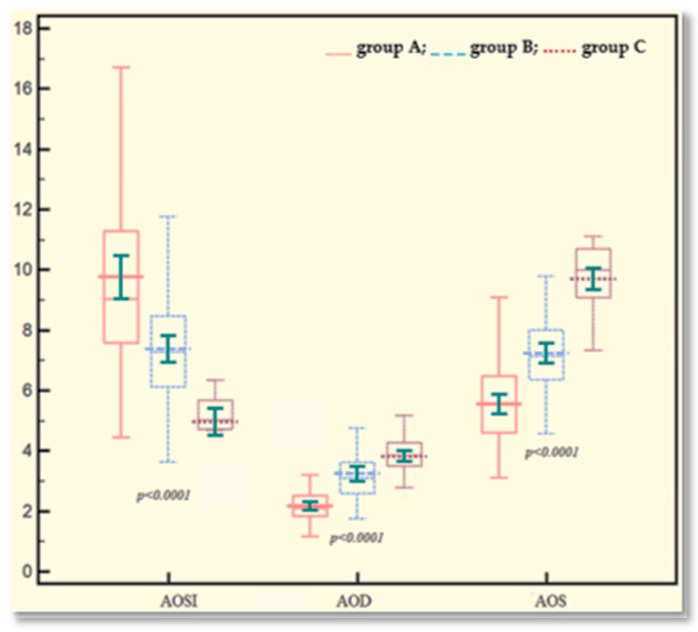
Distribution of E/e’ ratio, PWV values, AoSI, AoD and AoS in study groups. Legend: E/e´ ratio—ratio between the early diastolic velocity ascertained via pulse Doppler (E) and via tissue Doppler imaging (e’) at the level of the mitral annulus; PWV—pulse wave velocity; AoS—aortic strain; AoSI—aortic stiffness index β; AoD—aortic distensibility.

We assessed arterial and aortic elasticity and determined the parameters characterizing DD in both the patient groups and the controls, with the results presented in Table 3. We further compared data obtained from group A with those obtained in group B and analyzed the potential differences between the results obtained for group B and those obtained in controls, as shown in Table 3. The statistical distribution of PWV and E/e’ values and of the indexes characterizing aortic elasticity (namely AoSI, AoS, and AoD) in the three study groups is graphically represented in Figure 2. As can be observed in Table 3 and as expected, the increase in PWV values, which characterize AS, were significantly greater in patients with MS with a history of COVID-19 (group A) in comparison with group B (*p* = 0.0148), but even more significant differences were noted between both of the patient groups (A and B) and controls (*p* < 0.0001). In group A, only 18 patients had normal PWV values (≤9 m/s); thus, almost 70% of these patients had AS, whereas in group B, there were 21 subjects with normal PWV. Similar results were reported for AoS, AoSI β, and AoD. In group C, all participants had normal levels of both PWV and the indexes characterizing aortic elasticity. At 6 months, we evidenced normal PWV values (˂10 m/s) in 37 patients from group A and in 45 from group B; thus, there was a substantial reduction (with over 50%) of AS. In terms of aortic elasticity, we found similar differences regarding all studied indexes (AoS, AoSI β, and AoD) between patients with post-acute COVID-19 syndrome, both with and without MS (group A and B), with a *p* < 0.0001, but also between group B (without MS) and controls (*p* < 0.0001). Similarly, as in the case of AS, the 6 months’ evolution revealed a significant improvement of all these three indexes. 

Regarding the TTE-assessed parameters, as expected, both post-acute COVID-19 syndrome patient groups had worse LVEF values (thus, in normal range) than controls (*p* < 0.0001), but we evidenced a similar difference between patients with and without MS (*p* < 0.0001). Proceeding further to the analysis of the DD, we noticed that in group A, there were 42 subjects with DD, of which 25 patients could be considered to have type 1, 13 patients could be considered to have type 2, and the other 4 patients could be considered to have type 3 DD. By contrast, in group B, there were 12 patients with a DD (11 with type 1, and 1 with type 2). None of the controls showed any evidence of criteria suggesting a DD. By following the evolution of this abnormality, we noticed that over the course of three months, in group A, the number of affected patients decreased to 35, of which 24 had type 1, 9 had type 2, and another 2 had type 3 DD. At 6 months, 18 patients still had a DD (9 of type 1, 8 of type 2, and 1 with type 3). In group B, a similar progress was observed, so that at 3 months, 10 patients still had DD (9 had type 1 and one had type 2), whereas at 6 months, only 3 subjects still had DD (2 had type 1, and 1 had type 2). 

By analyzing the potential associations between AS, aortic elasticity, and some parameters defining DD and factors related to the severity of the SARS-CoV-2 infection, we evidenced powerful statistically significant correlations between the PWV, AoS, AoSI, E/e’, and TRV and the time elapsed since COVID-19 diagnosis, the amplitude of the infection described by the severity of the lung injury, the initial level of CRP, and the intensity of the post-acute COVID-19 syndrome, namely, the number of persistent symptoms, and the PCFS scale. Moderate statistically significant correlations were found with elements defining MS and the TyG index, and weak ones with the VAI and LAP, as presented in Table 4.

We started from the assumption that in individuals recovering from a SARS-CoV-2 infection, the magnitude of the acute immunological stimulation, which eventually occurs on a pre-existing pro-inflammatory profile, could influence both AS and aortic elasticity and, subsequently, DD. Thus, we employed multivariate linear regression analysis to identify the most significant independent predictors which influence PWV, AoS, AoSI, AoD, and E/e’. For this purpose, we built a regression model based on the forward stepwise method and employed the Akaike criteria to select the best model. Because factors such as age, BMI, TAS, TAD, and gender (the reason why we only selected female patients for our study) are already proven as important predictors for the development of AS, DD, and impaired aortic elasticity, to avoid their impact on our results, we excluded these from our analysis, considering them to be confounding factors. Consequently, we tested the following parameters as independent predictors for PWV, AoS, AoSI, and AoD: CRP, days since COVID-19 diagnosis, the severity of the lung injury, and PCFS, as well as TyG, VAI, and LAP. The best association for PWV, AoS, and AoSI was found with the time elapsed since the SARS-CoV-2 infection (β = −0.061, β = 0.03, β = −0.08, respectively, with *p* < 0.0001), followed by the magnitude of the pulmonary injury during the acute infection (β = −0.03, β = -0.03, β = 0.058, respectively, with *p* < 0.0001), the levels of CRP during the acute illness for PWV (β = 0.029, with *p* = 0.038), TyG index for AoS (β = −0.05, *p* < 0.0001), and VAI for AoSI (β = 0.596, *p* = 0.0001). For AoD, the most important predictors were not only the TyG index (β = −0.3, *p* < 0.0001), but also the severity of the acute infection, as expressed by CRP levels and the lung injury (β = −0.019, *p* = 0.01, β = −0.016, respectively; *p* = 0.003). No association was found for LAP and PCFS.

Regarding DD, the multivariate linear regression analysis was used only to identify the independent predictors for E/e’ values. As was the case in the previous analysis, age, BMI, TAS, TAD, and gender were excluded. The following parameters were tested: PWV, AoS, AoSI, AoD, CRP, lung injury, PCFS scale, days since diagnosis, TyG, VAI, and LAP. The model including AoS (β = −13.52 ± 11.31, *p* = 0.0062), CRP levels (β = 0.11 ± 0.015, *p* = 0.0001), and TyG values (β = 0.313 ± 0.115, *p* = 0.0074) was best associated with E/e’ values. 

## 4. Discussion

The occurrence of endothelial dysfunction as a consequence of a direct action of the SARS-CoV-2 virus on the ACE2 receptors of endothelial walls, but which is also modulated by precocious and delayed immunological effects, was observed from the early stages of the COVID-19 pandemic and was reported by several researchers [4,8,36,37]. In these circumstances, the impairment in arterial elasticity, which is expressed by an increase in AS and AoSI and a reduction in AoD and AoS, was a predictable consequence. It was concluded that these alterations will induce further cardiac dysfunctions, such as LVH and DD, as well as secondary injuries in other organs [17,38]. From the first months of the onset of COVID-19 in Europe, Schnaubelt et al. [39] reported impaired AS levels, as confirmed by elevated values of PWV, in infected people compared to healthy subjects and non-COVID-19 patients and associated this dysfunction with a worse clinical outcome [39]. Because accompanying comorbidities, such as systemic hypertension, ischemic heart disease, T2DM, obesity, and MS, which are also associated with accelerated vascular aging, are important contributors to the severity and clinical outcome of an infection with the SARS-CoV-2 virus, it was assumed that they would supplementarily lead to further degeneration of vascular walls, as highlighted in several studies [8,12,40]. Moreover, the SARS-CoV-2 virus also affects the micro-circulation, namely small vessels and capillaries, favoring the occurrence of DD. In particular, individuals with T2DM, obesity, and/or MS, who have an altered response of the innate immunity with an increased pro-inflammatory state, exhibit higher susceptibility to developing more severe injuries, a state which is conducive to enhanced severity of SARS-CoV-2 infection, as reported by Bruno et al. in their study [7]. The important contribution on the vascular function of previous chronic therapies (antihypertensive and anti-inflammatory drugs) but especially of drugs used to treat the acute infection (antiviral drugs, corticotherapy, immunomodulators, antibiotics, invasive respiration) should not be overlooked and has been debated in multiple studies [7,39]. 

Several studies have raised the issue that even in young, previously healthy individuals, vascular function would need a longer time to recover and, in some cases, it would never return to normal [18,41,42]. This phenomenon possibly explains some of the pathophysiological pathways responsible for the development of the post-acute and long COVID-19 syndromes. In this vein, in our study, we documented the increased prevalence of impaired AS, as assessed by PWV, but also of reduced AoD and AoS with an increased AoSI, which is associated with various patterns of DD in 121 female patients who developed post-acute COVID-19 syndrome during the recovery from a SARS-CoV-2 infection. Although we conducted this study on younger women (under 55 years old), all of whom were selected for being premenopausal and who considered themselves to be apparently healthy prior to this infection, at a careful examination, we realized that 67 of them fulfilled the diagnostic criteria for MS. We proceeded further to compare their results with those obtained in subjects without MS and with those obtained from an age-matched control group of healthy women who did not suffer from COVID-19 and in whom, as expected, the parameters characterizing AS, aortic elasticity, and DD were within normal limits. Surprisingly, PWV values, although significantly higher (*p* = 0.0148) in patients with post-COVID-19 syndrome and MS (group A) compared to those without MS (group B), were much more elevated than in individuals who did not suffer from this illness (*p* < 0.0001). This aspect has already been discussed by Schnaubelt et al. in their study [39]. Similar results were also observed for the parameters characterizing aortic elasticity and DD (*p* < 0.0001), an aspect that was also debated in other studies [43]. It is worth mentioning that by analyzing the existence of associations between these indexes and other factors characterizing SARS-CoV-2 infection, we observed the most powerful correlations with the time elapsed since the acute illness and its severity. Similar results suggest that, in a shorter time period, the severity of the COVID-19 illness and the time elapsed since the infection are more important predictors for the magnitude of AS, impaired aortic elasticity, and DD than the generic determinants of MS, as also reported in other articles [8,41,44,45]. The multivariate linear regression analysis employed in our study also revealed that the elements characterizing the acute illness were more powerful predictors for the development of AS, impaired aortic elasticity, and DD, followed by the TYG index as a surrogate for IR, the contribution of which was highlighted in numerous other significant studies [9,29,32,41].

Although at this moment, absolutely indisputable data over the evolution of these dysfunctions are still missing, several researchers have documented an improvement in parallel with the time elapsed since the SARS-CoV-2 infection [8,41,42,46]. Thus, there is a high probability that, especially in people with metabolic dysfunctions, such as MS, T2DM, and/or obesity, several of the pathophysiological mechanisms involved in vascular aging and degeneration that were exacerbated from COVID-19 infection could persist, leading to further cardiovascular injuries [47,48]. Therefore, because noninvasive assessment methods such as TTE and PWV are at our disposal, and the significance of the obtained measurements is unanimously accepted, it seems reasonable to employ them over other imagistic methods. These methods also have higher scores for early diagnosis and risk stratification during both the acute phase of the SARS-CoV-2 infection and the recovery and follow-up, especially in vulnerable categories of patients [19,41]. 

Study limitations: one of the limitations is represented by the small number of patients included in our study due to the difficulty of finding suitable subjects that could fulfill all of our inclusion/exclusion criteria. Another limitation was represented by the fact that we did not have a rigorous TTE assessment or an evaluation of AS preceding infection with the SARS-CoV-2 virus. The third limitation was the younger average age of four patients for the study of the alterations of arterial and aortic elasticity; however, this can be compensated for by our effort to avoid any potential influence exerted by gender, hormonal status, and age on these dysfunctions. 

## 5. Conclusions

Altered AS, reduced aortic elasticity, and DD are common findings among patients with post-COVID-19 syndrome, especially in those with a poor metabolic profile. The indexes characterizing those dysfunctions were significantly associated with the time elapsed since the acute illness and its severity, which was expressed by CRP levels and the extent of the lung injury, as well as by biological markers characterizing MS. Arterial and aortic stiffness, the initial levels of CRP, and the TyG index were identified by multivariate regression analysis as significant predictor factors for the development of DD.

## Figures and Tables

**Table 1:** Inclusion and exclusion criteria.

Inclusion Criteria	Exclusion Criteria
apparently healthy women, aged over 18, but younger than 55 years, able to sign the informed consent form; evidence of a recent mild/moderate infection with the SARS-CoV-2 virus, certified by a positive result of a RT-PCR assay of nasal and pharyngeal swabs, with a basic medical evaluation consisting of laboratory blood tests, ECG, and chest radiography or CCT scan; the availability of a recent medical evaluation (less than one year) attesting their satisfactory health status, without any significant cardiovascular diseases or prescription of therapies for various metabolic diseases, even if patients were occasionally found with elevated BBG, abnormal lipids panel, or were suffering from obesity or overweight, thus meeting the criteria for MS.	subjects not willing/not able to sign the informed consent; individuals aged over 55 years or postmenopausal, with an increased probability of suffering from an underlying significant cardiovascular condition; subjects who suffered from severe COVID-19 with certified cardiovascular complications or those with asymptomatic forms or without any medical evaluation throughout the acute phase of the illness; patients with already-confirmed cardiovascular diseases or following a treatment for a chronic disease or diagnosed during the initial assessment with significant cardiac dysfunctions; subjects without a recent medical assessment of their health condition.

**Table 2:** Clinical and laboratory results of the study groups.

Results of Clinical, Laboratory, and Echocardiographic Parameters	Group A: 67 Women with MS and a History of COVID-19	Group B: 54 Women without MS, But with a History of COVID-19	Group C: 40 Healthy, Age-Matched Women Controls	*p*		
				A–B	B–C	C–A
Mean age (years)	50.59 ± 4.53	47.76 ± 5.43	49.47 ± 5.14	0.0022	0.0126	0.2422
BMI (kg/m^2^)	30.1 (24.9–31.2)	24.8 (23.5–28.2)	24.6 (22.6–28.3)	<0.0001	0.4002	<0.0001
WC (cm)	90 (89–95)	79 (73–85)	75 (70.2–84)	<0.0001	0.2530	<0.0001
SBP (mmHg)	130 (120–135)	117.5 (100–120)	120 (120–130)	<0.0001	0.0007	0.0001
DBP (mmHg)	80 (70–80)	70 (60–70)	70 (70–75)	<0.0001	0.0114	0.0001
HR (b/min)	75 (75–80)	80 (75–85)	70 (70–75)	0.0042	0.0040	0.3063
Time elapsed since COVID-19 diagnosis	56 (56–70)	63 (56–70)	NA	0.0485	NA	NA
Initial lung injury (CCT)	15 (0–30)	0 (0–8)	NA	0.0002	NA	NA
Number of symptoms	6 (3–6)	3 (2.75–6)	NA	0.0011	NA	NA
PCFS level	2 (1–2)	1 (1–2)	NA	<0.0001	NA	NA
**Laboratory data**						
Initial CRP (mg/dL)	30.1 (25.6–32.5)	26.6 (12.1–30.2)	1.9 (1.2–2.6)	0.0051	<0.0001	<0.0001
BBG (mg/dL)	102 (100–113)	90 (88–94.2)	90 (87.25–96)	<0.0001	0.7860	<0.0001
LDL chol. (mg/dL)	130 (120–150)	100 (90–112.5)	100 (99.25–105.7)	<0.0001	0.2497	<0.0001
HDL chol. (mg/dL)	30 (30–40)	45 (40–51.25)	55 (50–60)	<0.0001	<0.0001	<0.0001
Triglycerides (mg/dL)	170 (160–180)	140 (130–145)	130 (120–140)	<0.0001	0.0122	<0.0001
Uric acid (mg/dL)	7.3 (7.1–7.8)	6.5 (6–6.8)	6.2 (5.9–6.4)	<0.0001	0.0035	<0.0001
eGRF (mL/min)	100 (100–110)	119 (110–121)	120 (115–125)	<0.0001	0.1276	<0.0001
TyG index	4.87 (4.8–4.9)	4.72 (4.67–4.75)	4.2 (4–4.3)	<0.0001	<0.0001	<0.0001
LAP	61.4 (55.7–75)	32.68 (22–43)	27.2 (19–39.2)	<0.0001	0.0363	<0.0001
VAI	4.35 (3.6–4.8)	2.38 (1.88–2.78)	0.6 (0.54–0.66)	<0.0001	<0.0001	<0.0001

Legend: MS—metabolic syndrome; BMI—body mass index; WC—waist circumference; SBP—systolic blood pressure; DBP—diastolic blood pressure; LDL chol—low-density-lipoprotein cholesterol; HR—heart rate; PCFS—post-COVID-19 functional scale; CRP—C-reactive protein; BBG—basal blood glucose; LDL—low density lipoprotein; HDL—high density lipoprotein; TyG—triglyceride–glucose index; VAI—visceral adiposity index; LAP—lipid accumulation product; T2DM—diabetes mellitus; MS—metabolic syndrome.

**Table 3:** Results of the PWV and TTE assessments of the study groups.

Results of PWV and TTE Parameters	Group A: 67 Women with MS and a History of COVID-19	Group B: 54 Women with a History of COVID-19, without MS	Group C: 40 Healthy, Age-Matched Women Controls	*p*		
				A–B	B–C	C–A
PWV (m/s)	10 (9–12)	10 (9–11)	7 (6–8)	0.0148	<0.0001	<0.0001
**Results of the transthoracic echocardiography**						
DAD (mm)	29.5 (28.8–30.3)	28.1(27.7–29.1)	28.4 (28–29)	<0.0001	0.5826	<0.0001
SAD (mm)	31 (30.7–32)	30 (30–31)	31.15 (30.8–31.9)	<0.0001	0.0001	0.9031
AoS (%)	5.6 (4.58–6.53)	7.14 (6.38–8)	10 (9–10.7)	<0.0001	<0.0001	<0.0001
AoSI β	9 (7.5–11.4)	7.27 (6.13–8.48)	5 (4.7–5.7)	<0.0001	<0.0001	<0.0001
AoD (10^−3^ mm Hg^−1^)	2.1 (1.83–2.52)	3.12 (2.6–3.7)	3.86 (3.5–4.28)	<0.0001	0.0001	<0.0001
LVEF (%)	55 (50–58)	60 (55–66.25)	65.5 (65–70)	<0.0001	<0.0001	<0.0001
LVMI (g/m^2^)	94.6 (88.6–98.7))	89.83 (75.37–94)	80.3 (70.14–88.3)	0.0002	0.0002	<0.0001
LAVI (mL/m^2^)	23.43 (19–33)	16.1 (14–21.38)	13.15 (12.7–13.7)	<0.0001	<0.0001	<0.0001
TRV (m/s)	2.68 (2.57–2.73)	2.54 (2–2.7)	1.3 (1–1.67)	0.0011	<0.0001	<0.0001
E/A ratio	0.98 (0.76–1.3)	1.11 (0.88–1.33)	1.27 (1.21–1.38)	0.2922	<0.0001	<0.0001
E/e’ ratio	14 (11.5–14.24)	11.66 (9.4–12.8)	8.98 (8.56–9.46)	<0.0001	<0.0001	<0.0001

Legend: PWV—pulse wave velocity; DAD—diastolic aortic diameter; SAD—systolic aortic diameter; AoS—aortic strain; AoSI—aortic stiffness index β; AoD—aortic distensibility; LVEF—left ventricular ejection fraction; LVMI—left ventricular mass index; LAVI—left atrial volume index; TRV—tricuspid regurgitation velocity; E/A—ratio between early and late diastolic velocity ascertained via pulse Doppler; E/e’ ratio—ratio between the early diastolic velocity ascertained via pulse Doppler (E) and in tissue Doppler imaging (e’) at the level of the mitral annulus.

**Table 4:** Correlation between parameters characterizing arterial and aortic stiffness and DD, and other clinical and laboratory factors in 121 patients (group A = B).

Parameter	PWV	AoS	AoSI	AoD	E/e’	TRV
Days since diagnosis	r = −0.66, *p* < 0.0001 95%CI [−0.753–(−0.551)]	r = 0.57, *p* < 0.0001 95%CI [0.444–0.685]	R = −0.58, *p* < 0.0001 95%CI [−0.690–(−0.452)]	R = 0.46, *p* < 0.0001 95%CI [0.314–0.595]	R = −0.5, *p* < 0.0001 95%CI [−0.621–(−0.350)]	R = −0.59, *p* < 0.0001 95%CI [−0.695–(−0.460)]
Lung injury	r = 0.63, *p* < 0.0001 95%CI [0.517–0.732]	r = −0.62, *p* < 0.0001 95%CI [−0.722–(−0.501)]	R = 0.6, *p* < 0.0001 95%CI [0.456–0.693]	R = −0.54, *p* < 0.0001 95%CI [−0.659–(−0.405)]	R = 0.6, *p* < 0.0001 95%CI [0.482–0.710]	R = 0.63, *p* < 0.0001 95%CI [0.513–0.730]
No. of symptoms	r = 0.63, *p* < 0.0001 95%CI [0.517–0.732]	r = −0.68, *p* < 0.0001 95%CI [−0.770–(−0.579)]	R = 0.58, *p* < 0.0001, 95%CI [0.451–0.689]	R = −0.55, *p* < 0.0001 95%CI [−0.666–(−0.417)]	R = 0.58, *p* < 0.0001 95%CI [0.452–0.690]	R = 0.69, *p* < 0.0001 95%CI [0.595–0.780]
PCFS	r = 0.56, *p* < 0.0001 95%CI [0.517–0.732]	r = −0.65, *p* < 0.0001 95%CI [−0.747–(−0.541)]	R = 0.56, *p* < 0.0001 95%CI [0.421–0.670]	R = −0.56, *p* < 0.0001 95%CI [−0.674–(−0.427)]	R = 0.62, *p* < 0.0001 95%CI [0.506–0.725]	R = 0.69, *p* < 0.0001 95%CI [0.589–0.766]
Initial CRP	r = 0.56, *p* < 0.0001 95%CI [0.419–0.668]	r = −0.59, *p* < 0.0001 95%CI [−0.699–(-.467)]	R = 0.54, *p* < 0.0001 95%CI [0.431–0.676]	R = −0.57, *p* < 0.0001 95%CI [−0.674–(−0.428)]	R = 0.72, *p* < 0.0001 95%CI [0.621–0.796]	R = 0.78, *p* < 0.0001 95%CI [0.702–0.873]
No. of MS factors	r = 0.41, *p* < 0.0001 95%CI [0.188–0.501]	r = −0.63, *p* < 0.0001 95%CI [−0.729–(−0.512)]	R = 0.52, *p* < 0.0001 95%CI [0.368–0.633]	R = −0.67, *p* < 0.0001 95%CI [−0.760–(−0.562)]	R = 0.5, *p* < 0.0001 95%CI [0.320–0.600]	R = 0.48, *p* < 0.0001 95%CI [0.335–0.610]
TyG index	r = 0.4, *p* = 0.001 95%CI [0.146–0.469]	r = −0.61, *p* < 0.0001 95%CI [−0.716–(−0.493)]	R = 0.5, *p* < 0.0001 95%CI [0.361–0.631]	R = −0.64, *p* < 0.0001 95%CI [−0.735–(−0.5200)]	R = 0.45, *p* < 0.0001 95%CI [0.295–0.581]	R = 0.4, *p* < 0.0001 95%CI [0.364–0.630]
VAI	r = 0.34, *p* = 0.0002 95%CI [0.158–0.478]	r = −0.53, *p* < 0.0001 95%CI [−0.653–(−0.396)]	R = 0.5, *p* < 0.0001 95%CI [0.356–0.625]	R = −0.60, *p* < 0.0001 95%CI [−0.708–(−0.480)]	R = 0.49, *p* < 0.0001 95%CI [0.348–0.619]	R = 0.44, *p* < 0.0001 95%CI [0.286–0.575]
LAP	r = 0.33, *p* = 0.0002 95%CI [0.167–0.484]	r = −0.56, *p* < 0.0001 95%CI [−0.678–(−0.434)]	r−0.47, *p* < 0.0001 95%CI [0.329–0.606]	R = −0.63, *p* < 0.0001 95%CI [−0.732–(−0.517)]	R = 0.5, *p* < 0.0001 95%CI [0.320–0.599]	R = 0.51, *p* < 0.0001 95%CI [0.366–0.632]
LVMI	r = 0.43, *p* < 0.0001 95%CI [0.282–0.572]	r = −0.54, *p* < 0.0001 95%CI [−0.663–(−0.411)]	R = 0.47, *p* < 0.0001 95%CI [0.324–0.602]	R = −0.54, *p* < 0.0001 95%CI [−0.661–(−0.408)]	R = 0.5, *p* < 0.0001 95%CI [0.354–0.623]	R = 0.45, *p* < 0.0001 95%CI [0.299–0.584]

Legend: LV-GLS—left ventricular global longitudinal strain; RV—GLS-right ventricular global longitudinal strain; E/e’—early mitral inflow diastolic velocity E to average e’ velocity (E/e’) in pulsed tissue Doppler; PT—pericardial thickness; BMI—body mass index; PCFS—post-COVID-19 functional scale; CRP—C-reactive protein; TyG—triglyceride–glucose index; VAI—visceral adiposity index; LAP—lipid accumulation product.

## Data Availability

Our data are available on Mendeley Data, V1, doi:10.17632/kh2xtvg9zy.1/ last accessed on 5 February 2023.
